# Two-dimensional myocardial deformation in coronary vasospasm-related Takotsubo cardiomyopathy: A case report of a serial echocardiographic study

**DOI:** 10.1097/MD.0000000000008232

**Published:** 2017-10-27

**Authors:** Ming-Jui Hung, Ta Ko, Chung-Yu Liang, Yu-Cheng Kao

**Affiliations:** a Section of Cardiology, Department of Internal Medicine, Chang Gung Memorial Hospital, Keelung; b Chang Gung University College of Medicine, Taoyuan, Taiwan.

**Keywords:** coronary vasospasm, speckle tracking echocardiography, Takotsubo cardiomyopathy

## Abstract

**Rationale:**

Although transient reduction in the left ventricular ejection fraction is characteristic of Takotsubo cardiomyopathy, little is known about the time-course changes of myocardial deformation in coronary vasospasm-related Takotsubo cardiomyopathy.

**Patient concerns:**

We retrospectively analyzed the time-course changes in left ventricle, right ventricle, and left atrium strain values in a patient with coronary vasospasm-related Takotsubo cardiomyopathy. We found that not only left ventricular strain but also left atrial strain was abnormal during acute Takotsubo cardiomyopathy due to coronary vasospasm. Right ventricular free wall strain was normal.

**Diagnoses:**

Coronary vasospasm-related Takotsubo cardiomyopathy.

**Interventions:**

A serial echocardiographic study.

**Outcomes:**

The left ventricular strain was still subnormal despite a normalized left ventricular ejection fraction 2 months later. The left atrial strain was normal when the left ventricular ejection fraction normalized.

**Lessons:**

From this limited experience, it is suggested that echocardiographic myocardial deformation analysis can provide more information than the standard ejection fraction in evaluating myocardial contractile function.

## 1 Introduction

Coronary vasospasm (CV) is a nitrate-responsive, spontaneously occurring angina accompanied by electrocardiographic ST-T changes or transient coronary artery occlusion.^[1]^ CV plays an important role in myocardial ischemic syndromes, including stable angina, acute myocardial infarction, life-threatening arrhythmias, and out-of-hospital cardiac arrest.^[2–4]^ CV has been suggested to be one of the causes of Takotsubo cardiomyopathy (TC).^[5]^ Most cases of TC occur transiently and are usually triggered by physical or psychological stress, although neurological disturbances have also been reported as triggers.^[6,7]^ Although transient reduction in the left ventricular ejection fraction is characteristic of TC, little is known about the time-course changes of myocardial deformation in CV-related TC.

## 2 Case report

**Figure 1:**
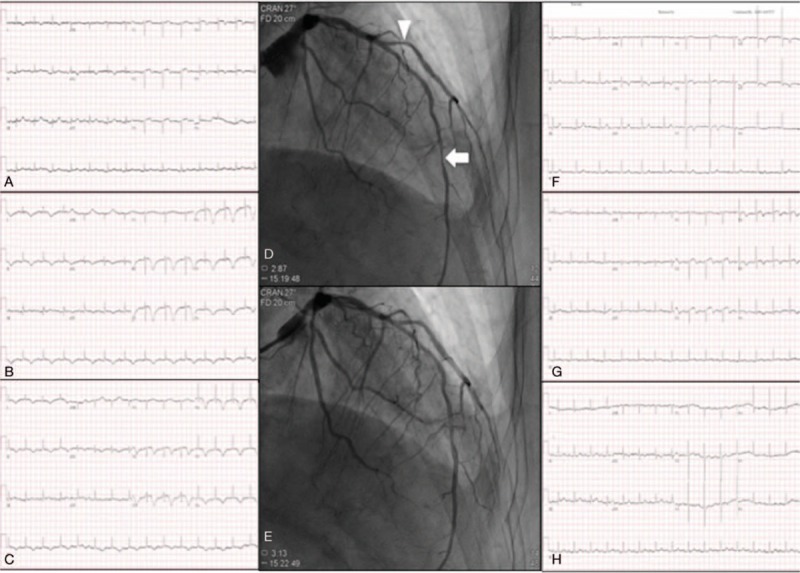
Electrocardiograms and coronary arteriograms. Serial electrocardiograms on day 1 (A), day 2 (B), and day 3 (C). Coronary arteriograms show provoked CV (D) in the middle portion of the left anterior descending coronary artery (arrow) and proximal portion of the largest diagonal artery (arrowhead) with decreased myocardial blush, which was relieved after intracoronary nitroglycerin 200 μg administration (E). Further resolution of electrocardiographic changes on the 8th day (F), 70th day (G), and 112th day (H). CV = coronary vasospasm.

We recently treated a patient with CV-related TC that was diagnosed by electrocardiography, 2-dimensional echocardiography, and coronary angiography. An 80-year-old woman was presented to our hospital with a 2-day history of chest pain. Her medical history included essential hypertension, type II diabetes mellitus, chronic kidney disease, and ischemic stroke without sequelae. She had been treated with amlodipine 5 mg, glimepiride 2 mg, and clopidogrel 75 mg once daily for the aforementioned well-controlled chronic diseases. Physical examination revealed a body mass index of 22.9 kg/m^2^ (body height of 160 cm and body weight of 58.6 kg), a heart rate of 82 beats/min, a respiratory rate of 18 breaths/min, and a blood pressure of 111/78 mm Hg. No cardiac murmur was detected and no other abnormalities were seen on physical examination. The hemogram showed mild normocytic anemia and biochemical test results revealed a troponin-I level of 4.916 ng/mL (reference, <0.5), a high-sensitivity C-reactive protein level of 19.373 mg/L (low risk, <1.0), a B-type natriuretic peptide level of 651 pg/mL (reference, <100), and a low-density cholesterol level of 115.6 mg/dL (reference, <100). Her 12-lead electrocardiogram showed ST-segment elevation in the anterior precordial leads and T-wave inversion in leads V_4–6_, I and aVL (Fig. 1A). The preliminary diagnosis was ST-segment elevation acute coronary syndrome. The patient declined primary coronary intervention because her chest pain was relieved soon after taking nitrate. She was subsequently admitted to the coronary care unit for further management.

**Figure 2:**
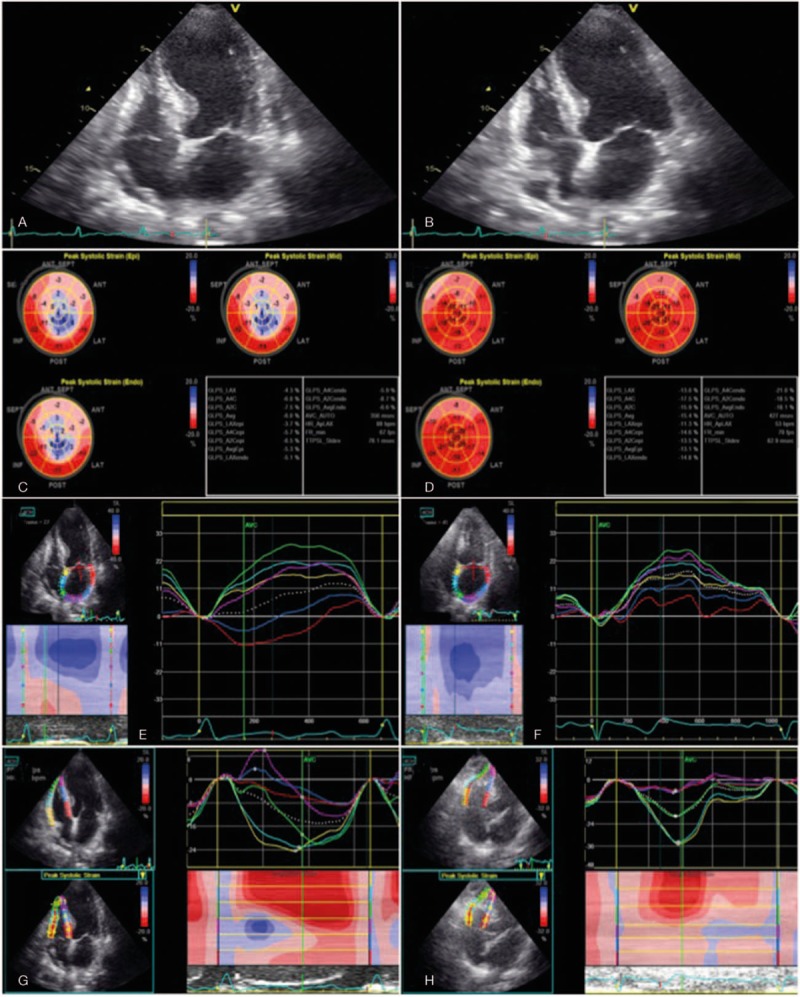
Echocardiograms: Two-dimensional echocardiograms show diffuse hypokinesis with normal contraction of the basal segments only at systole (A) compared with diastole (B). Myocardial deformation images: Left ventricular global longitudinal strain (C) and maximal left atrial strain (representative image from apical 4-chamber view, E) show reduced systolic strain on the 8th day, which improved on the 70th day (D and F, respectively). The right ventricular free wall strain values were within normal limits in both acute stage (G) and follow-up (H) studies. The averaged left atrial strain values are expressed as a white dashed line. The strain values of the 3 right ventricular free wall segments were averaged to obtain right ventricular free wall strain. LA = left atrium, LV = left ventricle, RA = right atrium, RV = right ventricle.

On the second hospital day, a follow-up 12-lead electrocardiogram revealed deep T-wave inversion in all leads with the exception of III, aVR, and V_1_ (Fig. 1B). On the third day, the electrocardiogram showed a reduction in T-wave inversion in the same leads (Fig. 1C). She did not experience chest tightness at that time. Coronary angiography performed on the fourth day and provocation testing revealed CV in the left anterior descending coronary artery (Fig. 1D and E) associated with decreased myocardial blush. Therefore, intracoronary nitroglycerin was administered with immediate relief of CV and increase of myocardial blush. The diagnosis of CV was made according to American College of Cardiology/American Heart Association Task Force guidelines for coronary angiography^[8]^ and isosorbide-5-mononitrate 30 mg twice daily was administrated for CV. The patient continued to complain of dyspnea on exertion and serial electrocardiographic studies showed atypical changes in ST-segment elevation (Fig. 1F), indicating acute ST-elevation myocardial infarction was less likely. A possible diagnosis of CV-related TC was considered. Therefore, on day 8 we performed transthoracic echocardiography to delineate possible CV-induced cardiac dysfunction. The left ventricular ejection fraction was estimated to be 26% with hyperkinetic basal segments and an apex aneurysm (Fig. 2A and B), typical features of TC. Based on the sequential symptoms of chest pain and dyspnea, the diagnosis was suggested to be CV-related TC. It was not likely that provocation testing for CV caused TC because of the short duration of coronary artery diameter reduction during provocation testing compared with prolonged CV-related myocardial ischemia-induced TC. Table 1 shows standard echocardiographic and 2-dimensional speckle-tracking echocardiographic data. Information on layer-specific left ventricular strain was obtained from 3 apical views and 3 parasternal short-axis views. Right ventricular free wall strain and left atrial strain were examined using an apical 4-chamber view and 3 apical views, respectively. All segmental values were averaged to obtain global strains. An institutional review board of Chang Gung Memorial Hospital approved the present study (no. 104-9129B). The results revealed a severely reduced left ventricular ejection fraction as well as reductions in longitudinal strain in the left ventricle (Fig. 2C), circumferential strain in the left ventricle and longitudinal strain in the left atrium (Fig. 2E). However, right ventricular systolic function and free wall strains were within the normal ranges (Fig. 2G). In addition, there was grade I diastolic dysfunction and a normal estimated left ventricular filling pressure estimated by septal E/e′ <15. The valve function was adequate without moderate/severe valvular dysfunction. Captopril 12.5 mg twice daily and furosemide 20 mg once daily were administered for systolic heart failure. The patient made a good recovery and was discharged uneventfully on the eighth day.

**Table 1:**
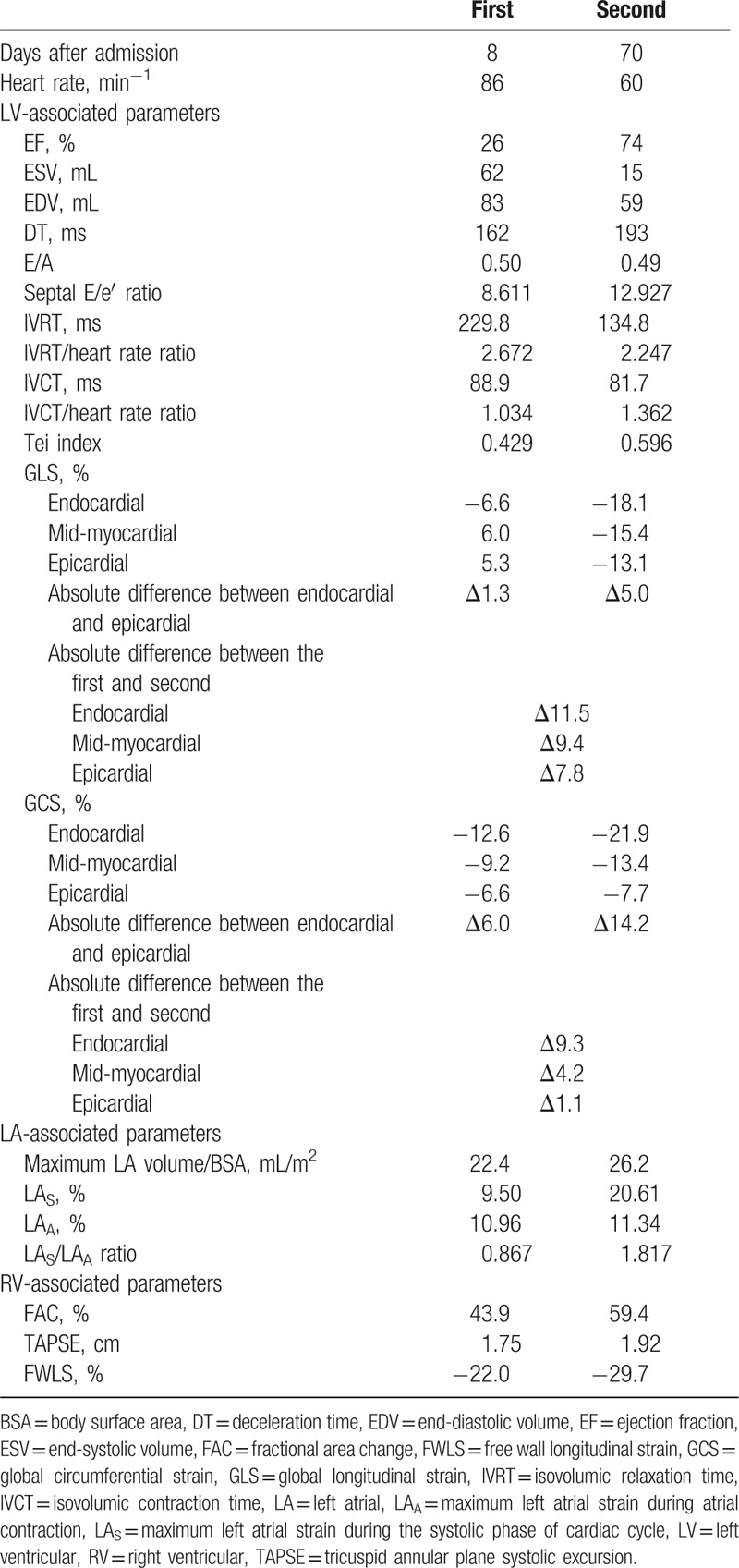
Echocardiographic data.

Follow-up 12-lead electrocardiograms on day 62 (Fig. 1G) and day 104 (Fig. 1H) after discharge from the hospital showed gradual resolution of the T-wave inversion. Follow-up echocardiography (Table 1) 2 months after discharge showed a normal left ventricular ejection fraction and improved but subnormal left ventricular longitudinal strain (Fig. 2D) and left circumferential strain.^[9]^ However, left atrial strain (Fig. 2F) was normalized and right ventricular free wall strain was stationary (Fig. 2H).^[10,11]^ The grade of left ventricular diastolic dysfunction was the same as the first examination and the estimated left ventricular filling pressure was not elevated. A final diagnosis of CV-induced TC was made based on the patient's history and electrocardiographic, echocardiographic, and coronary angiographic findings. We did not obtain cardiac biomarker levels during the follow-up period. However, these markers may correlate with prognosis and mortality during follow up.^[12]^ The patient was in stable condition at the most recent follow-up examination and continues to receive antispastic and anti-heart failure therapy in our cardiology outpatient clinic.

## 3 Discussion

The main findings of this report are as follows: Left ventricular strain was abnormal during acute TC with improvement and was still subnormal despite normalized left ventricular ejection fraction 2 months later, and left atrial strain was affected by CV-related acute TC. The abnormal left ventricular strain of TC is reasonable because many myocardial derangements, such as myocardial edema and electrocardiogram QTc interval prolongation, persist long after normalization of the left ventricular ejection fraction; however, maximum left atrial strain during the systolic phase of the cardiac cycle is also reduced in the presence of CV-induced acute TC. The endocardium usually undergoes larger dimensional changes than does the epicardium during systole.^[13]^ The contraction of the muscle fibers in the middle-wall, which is closely related to circumferential strain,^[14]^ better reflects intrinsic contractility than contraction of fibers in the endocardium. Consistent with these findings, our data showed slower improvement in the mid-myocardial and epicardial layers of circumferential strain than longitudinal strain. This indicates that the global left ventricular systolic function had not completely recovered even after the left ventricular ejection fraction had normalized. Normally, the maximum left atrial strain during left ventricular contraction is larger than that during left atrial contraction. The abnormal left atrial strain during left ventricular contraction indicates that left atrial systolic expansion is significantly less in acute TC than that in normal hemodynamic status. As expected, the magnitude of the left atrial strain improvement is larger than that of left ventricular strain because the atrium has a thinner muscular wall. Right ventricular involvement in TC with possible recurrence has been reported.^[15]^ It is suggested that excessive catecholamines can cause right ventricular stunning. In this reported case, the right ventricular free wall strain was not reduced possibly because of CV of the left anterior descending coronary artery rather than the right coronary artery. In the present study, the layer-specific left ventricular strain was used to express t time-course changes in the contractility of the 3 layers of the left ventricular myocardium. However, strain rate is a relative load-independent index of systolic function. Thus, peak systolic strain rate appears to be more useful than tissue Doppler velocity imaging to evaluate left ventricular dynamics during volume loading in patients with depressed left ventricular function.^[16]^ Further time-course studies of both ventricles and the left atrium in patients with TC induced by other causes are needed.

It is not easy to diagnose CV because it is reversible, often lasts for a few seconds to minutes, and is unpredictable. The unpredictability could be explained by the diversity of symptoms of CV, such as stable angina, acute coronary syndromes, life-threatening arrhythmias, out-of-hospital cardiac arrest, and even silent myocardial ischemia.^[2–4,17]^ In the present reported case, chest pain symptoms occurred before the development of heart failure, which is in line with some reports that CV may be a cause of heart failure.^[18–21]^ Strictly speaking, CV must be diagnosed based on coronary angiograms during an attack. However, it is not possible to perform coronary angiography during an attack and there is actually no need to do so. Provocation testing has been developed because it provides an opportunity to induce CV when patients are adequately prepared and monitored. Recently, the intracoronary route has been more favored in the catheterization laboratory because each coronary artery is examined separately and this method is associated with less systemic side effects from provocation drugs.^[22–24]^

Spastic coronary arteries have structural and functional changes. In a prior study using optical coherence tomography in patients with CV, spastic arterial segments were characterized by diffuse intimal thickening without calcium or lipid contents,^[25]^ similar to that seen in intravascular ultrasound studies.^[26,27]^ Recently, some investigators found intimal erosion and lumen irregularities on optical coherence tomography in patients with CV and acute coronary syndromes.^[28,29]^ Therefore, another form of atherosclerosis has been suggested in spastic arteries.^[30]^ The structural changes in the epicardial coronary arteries can be visualized using intracoronary imaging techniques; however, microvascular changes can be assessed indirectly such as with hyperemic microvascular resistance.^[31]^ Because CV decreases coronary blood flow causing myocardial ischemia, microvascular assessment provides insights beyond coronary flow velocity reserve and fractional flow reserve, which are more focused on epicardial stenosis. Recently, Yamanaga et al^[32]^ found impaired coronary microvascular resistance with preserved coronary flow velocity reserve and fractional flow reserve in patients with CV, indicating that the coronary relaxant impairment is present throughout the entire coronary tree instead of confined to the epicardial coronary segments. Based on the aforementioned studies, it is suggested that the pathogenic abnormalities of CV are present throughout the coronary arteries. Therefore, investigations in patients with angina and nonobstructive coronary artery disease should not only integrate the indicated intravascular ultrasound/optical coherence tomography and fractional flow reserve but also assess for functional vasomotor disorders using intracoronary provocation testing for CV.^[33]^

The etiology of TC is still elusive. Although CV was observed in prior reports,^[5]^ it cannot tell the whole story of TC. Because the clinical characteristics of TC include sudden, unexpected stress, and signs of sympathetic hyperactivation at presentation, catecholamines are suggested to have a central role in its development. The proposed mechanisms by which catecholamines cause myocardial stunning include vascular causes, such as epicardial CV; myocardial causes, such as intracellular calcium overload directly by catecholamines; and cardiac sympathetic nervous system causes, such as epinephrine-induced switch in signal trafficking in β2-adrenoreceptors,^[34,35]^ sympathetic nervous system hyperactivation, and disruption of local cardiac nerve terminals and norepinephrine spillover.^[36]^ There is increasing evidence that enhanced sympathetic stimulation plays an important role in the development of TC.^[37,38]^ Marfella et al^[39]^ found that apical myocardial ^123^I-metaiodobenzylguanidine uptake is impaired 14 days after acute TC. This finding supports the idea that local cardiac sympathetic nerve hyperactivation, disruption, and norepinephrine spillover cause TC. The important diagnostic investigations during acute TC include coronary angiography and radionuclide imaging. Urgent coronary angiography can show the anatomy and function of the coronary arteries. Myocardial scintigraphy can show myocardial perfusion at rest and during stress, and myocardial muscle metabolism. ^123^I-metaiodobenzylguanidine has been used to image myocardial nerve terminal activity. Similarly, it could be applied in follow-up of TC patients.^[40,41]^ Currently, no randomized controlled trials have been performed to demonstrate medical therapies for TC. However, heart failure medications, including beta-blockers, angiotensin-converting enzyme inhibitors or angiotensin receptor blockers, may be considered to prevent left ventricular remodeling. However, there is some debate regarding the effects of these heart failure medications on the recurrence rate and peripheral vascular resistance of TC.^[42]^ Because TC is characterized by involvement of the autonomic nervous system, therapeutic treatment targeting cardiac sympathetic dysfunction may be of crucial importance. Therapy with the antioxidant alpha-lipoic acid has been found to reduce oxidative stress and inflammation, resulting in improvement in adrenergic cardiac innervation, which may control complex molecular pathways involved in coronary flow distribution and consequently restore left ventricular segmental coronary blood flow.^[39]^

The present patient did not undergo endomyocardial biopsy because acute infective myocarditis was not likely. It is challenge to distinguish TC from acute infective myocarditis if there is evidence of acute myocardial edema and inflammation in a typical anatomical area, as is common during acute TC.^[43,44]^ TC can be considered a form of acute catecholaminergic myocarditis because of the appearance of T2-weighted short-tau inversion recovery on cardiac magnetic resonance and the rise of the cardiac troponin level. Evaluation of the patient's history, other clinical features, and close follow-up with imaging studies can help physicians differentiate these conditions (eg, the stressful trigger in TC versus the viral prodromal syndrome with fever in acute infective myocarditis).^[45,46]^

## 4 Conclusions

Myocardial deformation analysis can provide better information than standard echocardiography in evaluating the myocardium as the ejection fraction reflects ventricular geometric changes rather than the contractile function of the myocardium. Careful clinical, electrocardiographic, and echocardiographic follow-up studies are mandatory in TC patients to delineate myocardial function in addition to the standard ejection fraction.
